# Morphogenesis of Mammary Glands in Buffalo (*Bubalus bubalis*)

**DOI:** 10.1155/2014/687936

**Published:** 2014-04-27

**Authors:** Amit Challana, Anuradha Gupta, Neelam Bansal, Varinder Uppal

**Affiliations:** Department of Veterinary Anatomy, College of Veterinary Sciences, Guru Angad Dev Veterinary and Animal Sciences University, Ludhiana, Punjab 141004, India

## Abstract

The present research was elucidated on the morphogenesis of mammary gland of buffalo during prenatal development. Total of 16 foetuses ranging from 1.2 cm (34 days) to 108 cm CVRL (curved crown rump length) (317 days) were used for study. The study revealed that mammary line was first observed at 1.2 cm CVRL (34 days), mammary hillock at 1.7 cm (37 days), and mammary bud at 2.6 cm CVRL (41 days) foetuses. Epidermal cone was found at 6.7 cm CVRL (58 days) whereas primary and secondary ducts were observed at 7.4 cm CVRL (62 days) and 15 cm CVRL (96 days), respectively. Connective tissue whorls were reported at 18.2 cm CVRL (110 days) and internal elastic lamina and muscle layers at 24.1 cm CVRL (129 days). Lobules were observed at 29.3 cm CVRL (140 days), rosette of furstenberg at 39.5 cm CVRL (163 days), and keratin plug at 45.5 cm CVRL (176 days) foetus. Primordia of sweat and sebaceous glands around hair follicle were seen at 21.2 cm CVRL (122 days) of foetal life. Differentiation of all the skin layers along with cornification was observed at 69 cm (229 days) in group III foetuses.

## 1. Introduction

The latest trend in search of food leads to tremendous scope of livestock industry to develop breeds having more milk yield [1]. Milk is an excellent source of vitamins and minerals for the human diet [2]. The profit of livestock industry is directly related to maximum milk production from animals [1]. Optimizing the process of milk production will benefit consumers, producers, and the animals involved by increasing production efficiency and improving animal health [3].

India's status as the largest milk producing country in the world is attributed to its large bovine population comprising of 210.2 million cattle and 111.3 million buffaloes. Buffalo milk production in India is 62.4 million tonnes which is about 67.40% of total world (92.51 million tonnes) (FAOSTAT, 2010). Buffalo has been an integral part of livestock economy for over 5000 years providing draft power, milk, meat, and hides [4]. In India, buffaloes are preferred over cattle as a dairy animal and are eulogized as “the black gold of India” because of high milk fat content which fetches higher market price [5].

The global economic value of buffalo is tremendous and ultimately hinges on its mammary glands, either through its capacity to provide dairy products or because of its growth and production of milk which underlies successful animal production, welfare, and survival [6]. Milk yield and shape of lactation curve are determined by the number of mammary secretory cells and the secretory activity per cell [7].

During the lifetime of the animal the mammary gland probably undergoes more and greater changes in size, structure, composition, and activity than any other tissue or organ. These changes start during foetal life and continue even after the gland has reached maturity since it waxes and wanes during successive reproductive cycles [8]. The basic structures of the mammary glands are formed in foetal life and comprise a system of growing ducts that are confined to a very limited area around the teat or the gland [9].

The detailed histomorphological studies have been reported on morphogenesis of sheep [9] and goat [10–12], but scanty information is available on development of mammary gland at different stages of prenatal life in buffalo.

To improve efficiency of milk production, we must understand the mechanisms involved in mammary development stages of buffalo during foetal life. Hence, the morphogenesis of mammary tissue may reveal the ways to manipulate the development of mammary gland to enhance milk production in mature buffalo.

## 2. Materials and Methods

### 2.1. Collection of Samples

The present study was conducted on mammary glands of 27 buffalo foetuses. The foetuses of different gestational age were obtained from pregnant nondescript buffaloes slaughtered at Gazipur Slaughter House, New Delhi, and Veterinary Clinical Complex, GADVASU, Ludhiana. The tissue samples were collected after death/slaughter of the animals. The particulars of foetuses have been described in Table 1. After the collection, the foetal body length was measured as curved line in centimeter with the help of inelastic thread along the vertebral column between the most anterior part of frontal bone to the rump at ischiatic tuberosity and designated as crown rump length [13].

The approximate age of the foetuses was calculated by using the formula [14]

(1) $$\begin{array}{c} Y=28.66+4.496X\left(\text{CVRL}<20\;\text{cm}\right)\\ Y=73.544\;+\;2.256X\left(\text{CVRL}\geq 20\;\text{cm}\right), \end{array}$$

where *Y* is age in days and *X* is CVRL in centimeters.

Based on CVRL the foetuses were divided into three groups:

 group I: foetuses of CVRL between 0 and 20 cm;
 group II: foetuses of CVRL between 20 and 40 cm;
 group III: foetuses of CVRL above 40 cm.

### 2.2. Fixation and Processing

The small pieces of 1.0 cm thickness were collected for histomorphological studies. In small sized foetuses up to 2.6 cm CVRL the whole amount was taken as it is difficult to collect mammary glands at this age. In the foetuses from 6.7 to 110 cm CVRL teats with glandular portion were taken out and serial sectioning was done on the foetuses/mammary gland samples.

The tissue samples were fixed in 10% neutral buffered formalin (NBF) immediately after collection. Once the fixation was achieved, the tissues were processed for paraffin block preparation by acetone-benzene schedule [15]. The blocks were prepared and sections of 5-6 *μ*m thickness were cut and obtained on clean glass slides with rotary microtome. The paraffin sections were stained with Hematoxylin and Eosin stain [15] to study the morphogenesis.

## 3. Results 

Mammary gland develops from the ectoderm and mesoderm germ layers in the embryonic life. The cells differentiated to develop into the functional mammary gland occurred during the early foetal life.

### 3.1. Mammary Line

**Figure 1:**
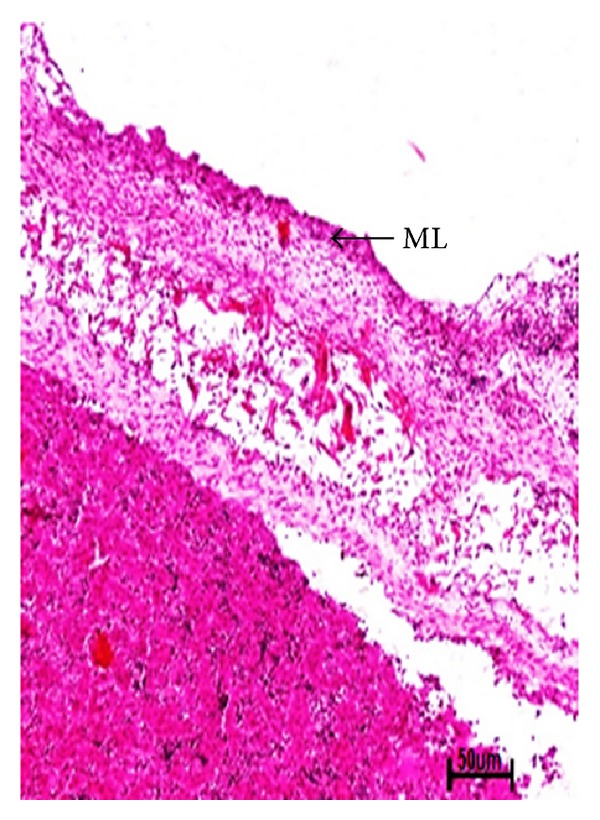
Photomicrograph of mammary line (ML) of buffalo foetuses in the ventral region at 1.2 cm CVRL (34 days). Hematoxylin and Eosin stain ×100.

**Figure 2:**
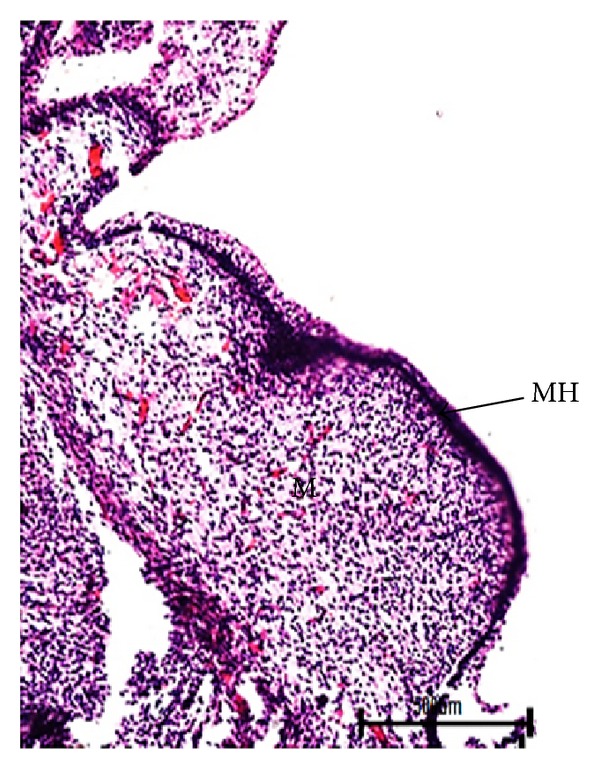
Photomicrograph of mammary hillock (MH) of buffalo foetuses at 1.7 cm CVRL (37 days). Hematoxylin and Eosin stain ×100.

The earliest recognizable stage of mammary gland development in the present study was mammary line found in 1.2 cm CVRL (34 days) buffalo foetus on the ventral side in the inguinal region posterior to umbilicus (Figure 1). The proliferation of mesenchymal cells resulted in condensation of mammary line into cone shaped mammary hillock at 1.7 cm CVRL (37 days) of foetal age (Figure 2).

### 3.2. Mammary Bud

**Figure 3:**
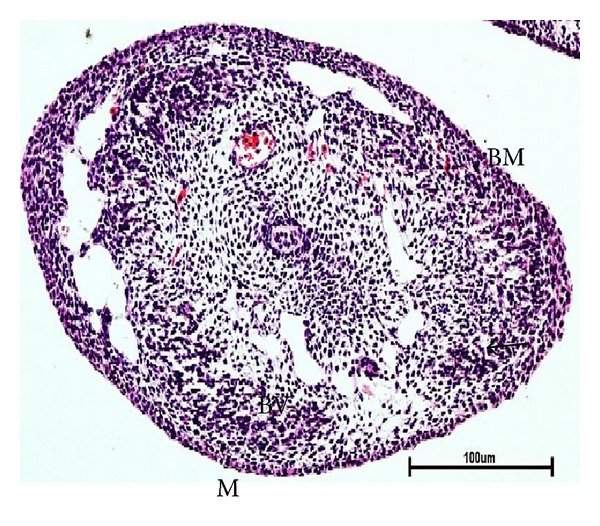
Photomicrograph of mammary bud of buffalo foetuses showing concentric layers of mesenchymal cells and blood vessel (BV) lined by basement membrane (BM) at 2.6 cm CVRL (41 days). Hematoxylin and Eosin stain ×100.

The mammary bud is the most prominent stage of mammary embryonic development in buffaloes. It was observed in 2.6 cm CVRL (41 days) buffalo foetus (Figure 3). It was ovoid in shape with its long axis perpendicular to the surface of the foetus.

### 3.3. Primary Sprout

**Figure 4:**
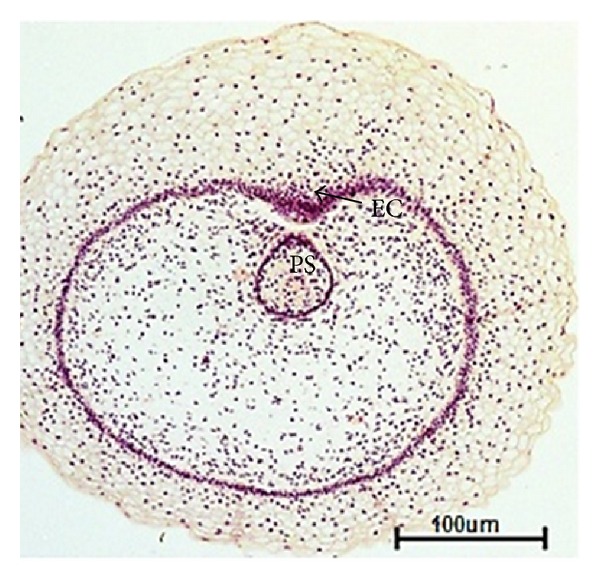
Photomicrograph of primary sprout (PS) and epidermal cone (EC) at 6.7 cm CVRL (58 days). Hematoxylin and Eosin stain ×40.

**Figure 5:**
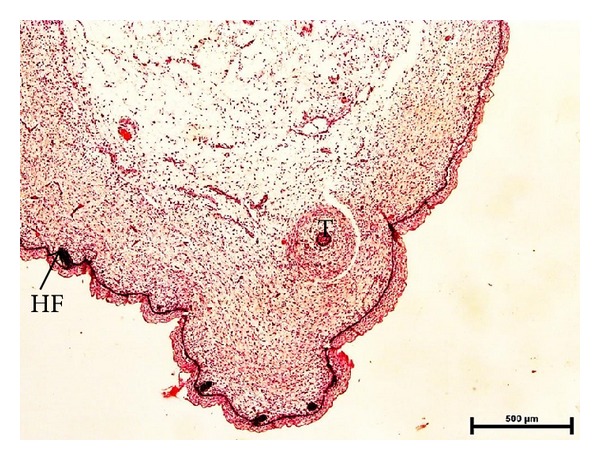
Photomicrograph of initiation of teat development along with epidermis, dermis, gland sinus, and hair follicle (HF) at 7.4 cm CVRL (62 days). Hematoxylin and Eosin stain ×40.

**Figure 6:**
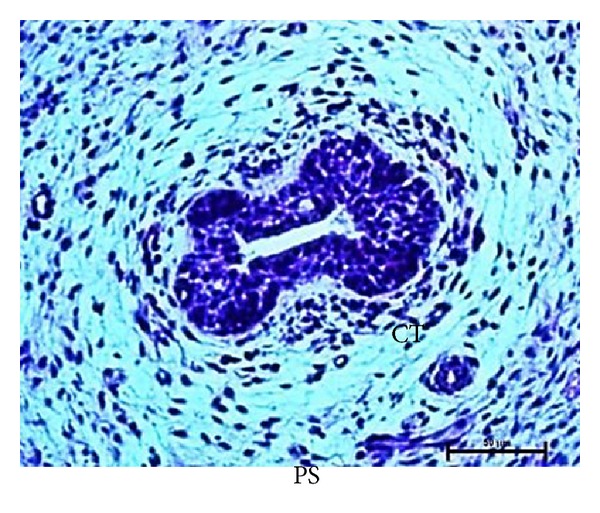
Photomicrograph of luminized primary sprout (PS) surrounded by connective tissue (CT) at 12.6 cm CVRL (85 days). Hematoxylin and Eosin stain ×400.

The mesenchymal cells surrounding the mammary bud apparently proliferated to form the ventral projection of mammary tissue which developed to epidermal cone (primitive teat) at 6.7 cm CVRL (58 days) in the inguinal region of buffalo foetus (Figure 4). The epidermal cells of the bud presumably were elongated by rapid cell proliferation into the underlying mesenchymal tissue along the length of the teat to form primary sprout at 7.4 cm CVRL (62 days) of foetal age (Figure 5). These cells got extended as cellular chords and entered deep into the base of teat at 10.7 cm CVRL (77 days) of foetal age. At 10.7 cm CVRL (77 days), luminization of the primary duct was observed in the area of primary sprout leading towards the teat. The luminization of primary sprout towards the apex of the teat on the distal end and towards the gland sinus proximally was observed in 12.6 cm CVRL (85 days) of buffalo foetus. The primary duct gave rise to the teat cistern, gland cistern, and major duct system of mammary gland (Figure 6).

### 3.4. Secondary Sprout

**Figure 7:**
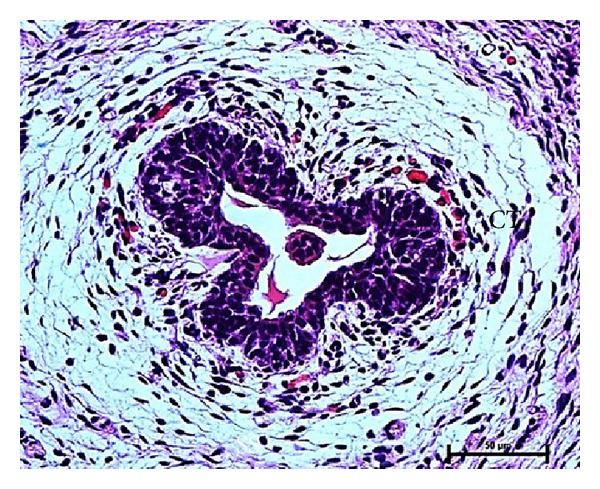
Photomicrograph of secondary sprout (SS) at 15 cm CVRL (96 days). Hematoxylin and Eosin stain ×400.

**Figure 8:**
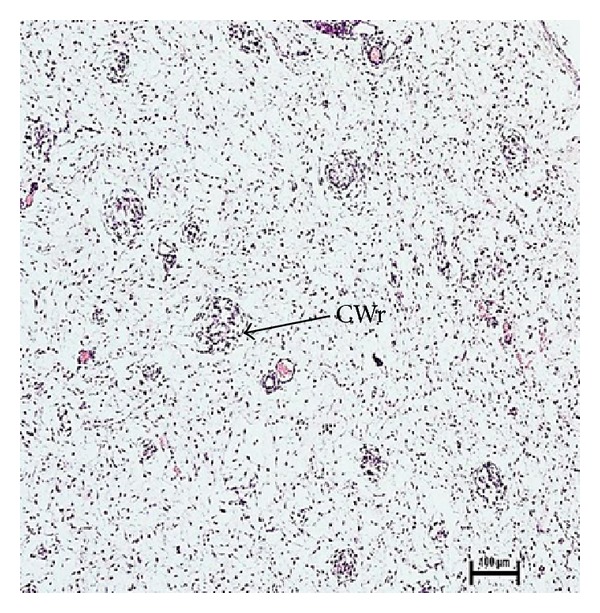
Photomicrograph connective tissue whorl (CWr) at 18.2 cm CVRL (110 days). Hematoxylin and Eosin stain ×100.

The secondary ducts appeared as irregular shaped hollow sacks at 15 cm CVRL (96 days) of foetal age (Figure 7). These ducts would form the duct system of the mammary gland. The lumen of secondary ducts began to develop by cellular degeneration of epithelial cells of primary sprout at various angles into the surrounding mesenchyme at 18.2 cm CVRL (110 days) (Figure 8).

The secondary ducts gave rise to tertiary branches at 21.2 cm CVRL (122 days) and further branching of the tertiary ducts was observed at 24.1 cm CVRL (130 days) that proceeded towards the fat pad.

### 3.5. Teat Formation

As the age advanced, gradually the epidermal cone got elevated along the mammary bud and the development of teat was initiated at 6.7 cm CVRL (58 days). With increasing age, the length of teat also increased.

### 3.6. Fat Pad

The mesenchymal cells at the base of developing mammary gland differentiated into fat pad at 45.5 cm CVRL (176 days) of buffalo foetus.

### 3.7. Gland Cistern

The initiation of gland cistern was observed in 15 cm CVRL (96 days) buffalo foetus. The continual growth of the lumen of the primary sprout pushed back the cells lining the primary sprout towards glandular tissue of developing mammary gland resulting in formation of fully developed gland cistern at 47.8 cm CVRL (181 days) in buffalo foetuses (Figure 10).

### 3.8. Teat Cistern

The canalization of primary sprout proceeded downward and initiated the formation of teat cistern in 15 cm CVRL (96 days) of buffalo foetus. Well distinct teat cistern was noticed at 21.2 cm CVRL (122 days). The lumen of teat cistern continued downward to form teat canal at 21.2 cm CVRL (122 days) of buffalo foetus.

**Figure 9:**
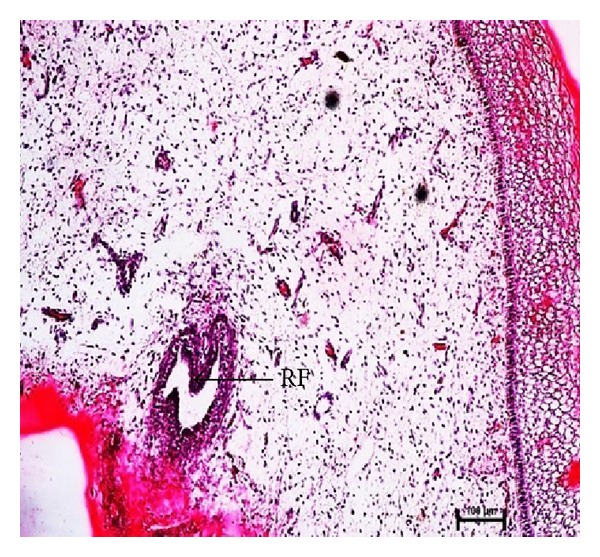
Photomicrograph of rosette of furstenberg (RF) at 39.5 cm CVRL (163 days). Hematoxylin and Eosin stain ×100.

**Figure 10:**
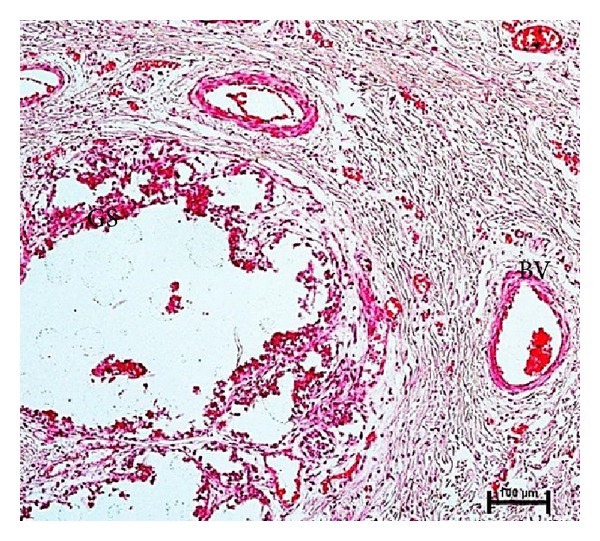
Photomicrograph of gland sinus (GS) surrounded by blood vessels (BV) at 47.8 cm CVRL (181 days). Hematoxylin and Eosin stain ×100.

**Figure 11:**
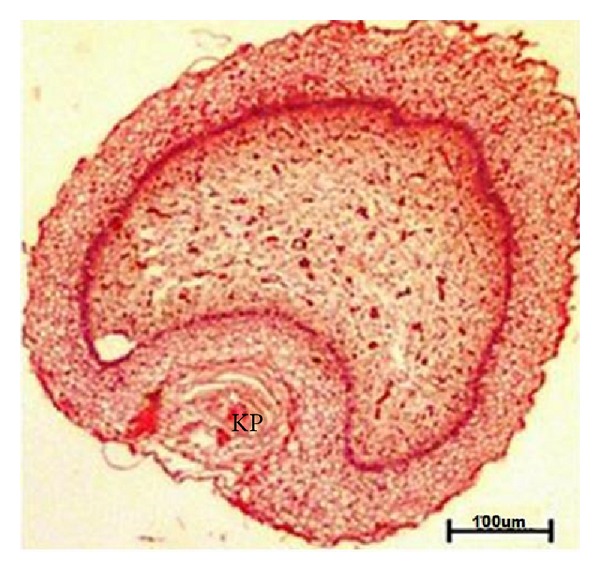
Photomicrograph of keratin plug at tip of teat in 45.5 cm CVRL (176 days). Hematoxylin and Eosin stain ×40.

The rosette of furstenberg was observed at 39.5 cm CVRL (163 days) at junction of teat canal and streak canal (Figure 9) and keratin plug was found at 45.5 cm CVRL (176 days) of buffalo foetuses (Figure 11).

### 3.9. Microvascularization

The blood vessels were observed in the mesenchymal tissue of mammary bud at 2.6 cm CVRL (41 days) of buffalo foetus. At 18.2 cm CVRL (110 days), nerves and small blood vessels were found in close association with connective tissue whorl (Figure 8).

With advancement of foetal age the numerous blood vessels were seen around secondary sprout, developing gland, and teat cistern. At 69 cm CVRL (229 days), blood and lymphatic vessels along with nerve bundles were present throughout the mammary gland.

### 3.10. Skin and Its Appendages

**Figure 12:**
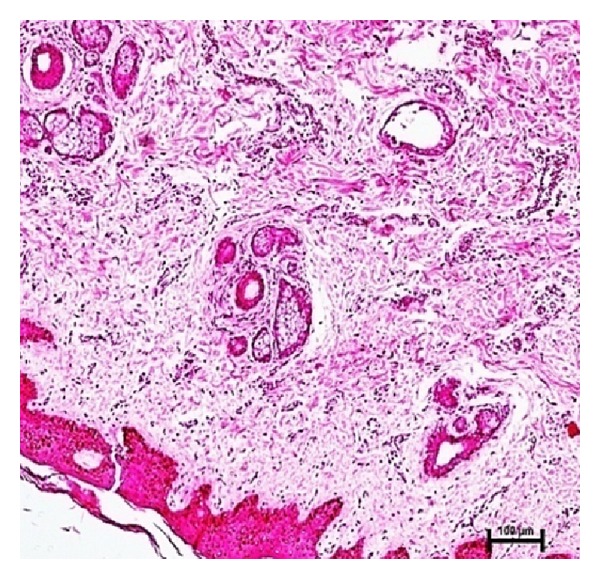
Photomicrograph of hair follicle surrounded by sebaceous gland and sweat glands at 82 cm CVRL (259 days). Hematoxylin and Eosin stain ×100.

The skin was comprised of distinct epidermis and dermis at 6.7 cm CVRL (58 days) buffalo foetuses. Epidermis was 4-5 cell layers thick at 6.7 cm CVRL (58 days) which was increased to 7-8 cell layers at 7.4 cm CVRL (62 days) and 12–14 at 10.7 cm CVRL (77 days). With advancement of fetal age thickness of epidermis decreased and became 4-5 cell layers at 18.2 cm CVRL (110 days). Dermis was clearly divided in two layers at 18.2 cm CVRL (110 days). Outer layer that is towards epidermis was loosely arranged, whereas the inner layer was dense. The thickness of dermis increased with advancement of fetal age. Primordia of hair follicle were observed in 6.7 cm CVRL (58 days) buffalo fetus. Primordia of sweat and sebaceous glands were found at 21.2 cm CVRL (122 days) of buffalo fetus. From 69 cm CVRL (days) onwards, epidermis was fully developed as in adult at streak canal and around mammary gland (Figure 12).

## 4. Discussion

The development of the mammary gland is a continuous process throughout life that begins when the animal is a foetus and continues through adulthood [16]. It starts as thickening of ectodermal cells on the ventral surface of the embryo giving rise to the mammary band. As proliferation of the ectoderm cells and mesenchymal cells continues, the mammary band develops into mammary streak, mammary hillock, and ultimately into mammary bud.

Mammary band developed at 9 mm CRL in sheep, 5 mm CRL in goat, and 14 mm CRL in cattle [17]. Similarly, [18, 19] observed that the mammary gland formed from an invagination of ectoderm at 1.4 cm CVRL in cow embryo. The mammary band runs along both sides of the midline in the inguinal region at day 40 in sheep foetuses [9].

Mammary line appeared in the inguinal region on either side of midventral line, medial to thigh at 38 days (1.0 cm CRL) in buffalo foetuses [20], whereas Singh (2000) observed four mammary anlages on the ventral abdominal wall caudal to umbilicus between the hind limbs in buffalo foetus at 90–109 days of gestation.

The mammary line was observed in the inguinal area posterior to umbilicus at 40 days in sheep foetuses [9]. The mammary line extended from the forelimb buds to the hind limb buds at 30 days of gestation in bovine foetus [21]. The median line was observed medial to thigh in 4.50 cm CRL and was discoid shaped mammary crest in 5.00 cm CRL goat foetuses [11].

Mammary bud was found at 3.0 cm CRL in cow foetuses [18]. The mammary bud was most often elongated and ovoid in shape in cattle embryo [22].

The mammary hillock and bud were the prominent stages of mammary embryonic development in ruminants [19]. In buffalo and bovine foetuses mammary bud was noticed at 55 days (4.20 cm CVRL) and 43 days, respectively [20, 23], whereas [9] mammary bud was found at 40 days in sheep foetuses (Table 2). However, the mammary bud lengthened and branched from day 58 onwards in bovines [24].

Formation of mammary bud was observed between 4.4 and 6.0 cm CRL (44–49 days) and the cells of the mammary bud progressed deeper into the dermis at 7.9 cm CRL in goat foetus [25].

The primary duct was found at 12.00 cm CRL in cow foetuses [18]. It got elongated and entered deep into the teat base at 102 days (14.30 cm CRL) [20]. The luminization progressed towards the distal end of the duct near the teat apex in 120 days (18.50 cm CRL) buffalo foetuses. The canalization of primary duct was found at 120 to 146 days in buffalo foetuses [26].

The primary duct was observed at day 80 in bovine [23] and at 15.0 cm CRL (60 days) in sheep foetus [9]. It sunk deeper into the teat base at 12.5 cm CRL (64 days) in goat foetuses [25]. The primary duct was reported at 11.50 cm CRL in goat foetuses [11]. This structure got extended as a cellular cord and entered deep into the base of the teat at 12.50 cm CRL.

Numerous secondary ducts were found at 80 days of sheep foetuses [9, 27], at 254 days in buffalo foetuses [26], and at 13.8 cm CRL (68 days) in goat foetuses [25]. The secondary ducts were budding off at different angles to the proximal end of the primary duct in various directions between 14 and 17 cm CRL (69–78 days) in goat foetuses.

Development of teat was found at 80 days of gestation in bovine foetuses [28]. The development of teat was initiated by an elevation of epidermal cone surrounding the mammary bud at 5.70 cm CRL in buffalo fetuses [20], whereas [11, 25] a papilla was observed like teat discernible on either side of midline in inguinal region between two thighs at 9.5 cm CRL (58 days) and 6.70 cm CRL, respectively, in goat foetuses.

The fat pad was found at day 140 and day 80, respectively, in sheep foetuses [9, 27]. Reference [26] found fat pad at 120 days of foetal life in buffalo. The latter was observed on the base of the developing mammary gland at 180 days of gestation in cattle [21]. The fat pad was reported at 19.5 cm and 20.0 cm CRL, respectively, in goat foetuses [11, 25].

Gland cistern was found at 17.00 to 21.00 cm CRL in cow foetus and [9, 18] found the gland cistern near the proximal end of primary duct at day 80 in sheep foetuses. All the ducts were luminized at 38.6 cm CRL (139 days) [25], whereas gland cistern between the fat pads was observed at 17.60 cm CRL in goat foetuses [11].

The streak canal was the distal most part of the primary duct that canalised at last but lumen was not fully formed up to 152–182 days in buffalo foetuses [26]. The streak canal and teat cistern were reported at 100 days in bovine foetuses [23] and at 80 days in sheep foetuses [9].

Teat cistern was described at 120 days of gestation in mammals [21, 24]. The teat canal was found at 21.7 cm CRL (91 days) in goat foetuses [25].

Nerves and small blood vessels formed in close association with each bundle of developing adipose tissue at day 80 and elastic fibres at day 90 in sheep foetuses [9]. The numerous blood capillaries invaded the bud at 4.4 cm to 6.0 cm CRL (44−49 days) and 7.20 cm CRL in goat foetuses, respectively [11, 25].

The distinct epidermis and dermis were observed between 74 and 115 days of gestation in buffalo [29]. The epithelium was divided into 4 layers on stratum germinativum, stratum granulosum, stratum intermedium, and stratum basale goat foetus [30], whereas distinct dermis was reported between 74 and 115 days of gestation in buffalo foetuses.

The first evidence of hair follicle was found at 102 days in buffalo foetuses [31, 32]. The hair follicles began to develop around the 120th day of gestation in bovines [21]. The hair follicle was observed on the teat at 65 days in goat foetuses [12].

The sweat gland was found at 140–150 days buffalo foetuses [29]. The primordium of sweat glands was observed at 139 days in buffalo foetus [31]. The sweat glands were seen for the first time at 83 days in goat foetuses [12].

The sebaceous glands were reported at 158 days in buffalo foetuses [31]. It was associated with each primary follicle at 120 days in sheep foetuses [9]. A bunch of sebaceous glands were reported around the hair follicles in 117–139 days in goat foetuses [12].

## Figures and Tables

**Table 1:** Details of buffalo foetuses used in study.

Sr. number	Group	Animal number	CVRL (cm)	Estimated age (days)
1	**Group I**	A1	1.2	34
2	-do-	A2	1.7	37
3	-do-	A3	2.6	41
4	-do-	A4	6.7	58
5	-do-	A5	7.4	62
6	-do-	A6	10.7	77
7	-do-	A7	12.6	85
8	-do-	A8*	15	96
9	-do-	A9	18.2	110
10	**Group II**	B1*	20.1	119
11	-do-	B2	21.2	122
12	-do-	B3	24.1	130
13	-do-	B4	29.3	140
14	-do-	B5*	32	146
15	-do-	B6	39.5	163
16	**Group III**	C1*	43	172
17	-do-	C2	45.5	176
18	-do-	C3	47.8	181
19	-do-	C4*	58	204
20	-do-	C5	69	229
21	-do-	C6*	74	241
22	-do-	C7	82	259
23	-do-	C8	89.5	275
24	-do-	C9*	94	286
25	-do-	C10	102	303
26	-do-	C11	108	317
27	-do-	C12	110	320

The samples marked by “∗” have been collected from Veterinary clinics, GADVASU Ludhiana.

**Table 2:** Summary of morphogenesis in buffalo foetal mammary gland.

Sr. number	Structure developed	CVRL (cm) buffalo	Age (days) buffalo	CRL (mm) cattle	Age (days) cattle
1	Mammary line	1.2	34	17	35
2	Mammary hillock	1.7	37	21	40
3	Mammary bud	2.6	41	25	43
4	Epidermal cone and primary sprout	6.7	57	—	—
5	Developed teat	7.4	62	80	65
6	Luminized primary sprout	10.7	77	120	80
7	Secondary sprout	15	96	160	90
8	Connective tissue whorls	18.2	110	—	—
9	Teat cistern and internal elastic lamina	24.1	128	300	130
10	Lobules and interlobular septa	29.3	140	—	—
11	Rosette of furstenberg	39.5	163	—	—
12	Keratin plug, fat pad, and gland cistern	45.5	176	—	—
13	Well-developed Teat canal	47.8	181	—	—
14	Well-developed sweat and sebaceous gland	82	258	—	—

Jenkinson (2003)[9].
